# Advanced Hydrogels for Cartilage Tissue Engineering: Recent Progress and Future Directions

**DOI:** 10.3390/polym13234199

**Published:** 2021-11-30

**Authors:** Mahshid Hafezi, Saied Nouri Khorasani, Mohadeseh Zare, Rasoul Esmaeely Neisiany, Pooya Davoodi

**Affiliations:** 1 Department of Chemical Engineering, Isfahan University of Technology, Isfahan 84156-83111, Iran; mahshid.hafezi96@pa.iut.ac.ir; 2 School of Metallurgy and Materials, University of Birmingham, Birmingham B15 2TT, UK; m.zare@bham.ac.uk; 3 Department of Materials and Polymer Engineering, Faculty of Engineering, Hakim Sabzevari University, Sabzevar 96179-76487, Iran; r.esmaeely@hsu.ac.ir; 4 School of Pharmacy and Bioengineering, Hornbeam Building, Keele University, Staffordshire ST5 5BG, UK; 5 Guy Hilton Research Centre, Institute of Science and Technology in Medicine, Keele University, Staffordshire ST4 7QB, UK

**Keywords:** polymeric hydrogels, self-healing, articular cartilage, tissue engineering

## Abstract

Cartilage is a tension- and load-bearing tissue and has a limited capacity for intrinsic self-healing. While microfracture and arthroplasty are the conventional methods for cartilage repair, these methods are unable to completely heal the damaged tissue. The need to overcome the restrictions of these therapies for cartilage regeneration has expanded the field of cartilage tissue engineering (CTE), in which novel engineering and biological approaches are introduced to accelerate the development of new biomimetic cartilage to replace the injured tissue. Until now, a wide range of hydrogels and cell sources have been employed for CTE to either recapitulate microenvironmental cues during a new tissue growth or to compel the recovery of cartilaginous structures via manipulating biochemical and biomechanical properties of the original tissue. Towards modifying current cartilage treatments, advanced hydrogels have been designed and synthesized in recent years to improve network crosslinking and self-recovery of implanted scaffolds after damage in vivo. This review focused on the recent advances in CTE, especially self-healing hydrogels. The article firstly presents the cartilage tissue, its defects, and treatments. Subsequently, introduces CTE and summarizes the polymeric hydrogels and their advances. Furthermore, characterizations, the advantages, and disadvantages of advanced hydrogels such as multi-materials, IPNs, nanomaterials, and supramolecular are discussed. Afterward, the self-healing hydrogels in CTE, mechanisms, and the physical and chemical methods for the synthesis of such hydrogels for improving the reformation of CTE are introduced. The article then briefly describes the fabrication methods in CTE. Finally, this review presents a conclusion of prevalent challenges and future outlooks for self-healing hydrogels in CTE applications.

## 1. Introduction

Cartilage defects as a result of aging and degenerative pathology, sports-related injuries, unexpected events, fatness, diseases, etc. have been noticed for more than 200 years. Non-vascular and finite cellular tissue of cartilage causes its limited regeneration [1]. With the growth of the elderly population in recent years, it is predicted that more than 15% of people aged 60 years and older (nearly 310 million people) will develop cartilage-related problems [2]. Although surgical methods, such as cartilage chondroplasty and microfractures, have been developed to treat cartilage defects, they have been unable to entirely repair the damaged cartilage. The current restrictions of cartilage surgery, such as complicated surgical procedures, post-infection, risk of the immune response, and poor-quality of the regenerated cartilage, have created a research field in tissue engineering and biological sciences to advance new cartilage tissue treatments [1,2,3]. However, the important constraint limiting CTE outcomes is the poor cell migration and growth inside implanted scaffolds, which yields new cartilage with undesirable physiological properties [2,3].

Hydrogels are appearing as an attractive class of biomaterials for organ regeneration and tissue repair due to interesting properties including tunable elasticity and stiffness, high-water content (typically 70%–99%), excellent biocompatibility, biodegradation, etc. Their three-dimensional (3D) network structures are made of natural macromolecules and/or synthetic polymers upon physical/chemical cross-linking [4]. The mechanical strength of the natural hydrogel scaffolds can range from 0.45 to 5.65 MPa [4] while synthetic hydrogels could attain 15–125 MPa [4]. In recent years, hydrogels for various biomedical applications have been prepared via the blending of both natural and synthetic polymers, thereby permitting the regulation of the physical and chemical characteristics of final products to meet their ultimate application [5]. Hydrogel properties can also be modified through chemical functionalization and physical manipulation (e.g., scaffolding) to mimic physicochemical and biological properties desired for a particular tissue construct [4,5].

Self-healing ability is one of the interesting properties of native tissues to repair injuries. Due to tensions and stresses exerted during physical activities, implanted hydrogels usually experience microcracks and structural defects. These microcracks gradually grow in size and finally yield to failure of the hydrogel structure [6]. Tissue engineering has taken a novel path for the regeneration of cartilage by using self-healing hydrogels. Self-healing hydrogels offer unique benefits such as self-repairing of damages, retaining structural integrity, and long-term functionality [7,8,9]. Despite many similarities between synthetic self-healing hydrogels and the extracellular matrix (ECM), hydrogels have demonstrated some drawbacks such as insufficient mechanical strength, low fracture energies (<15 J m^−2^), low cell viability, etc. [10]. As presented by some researchers, self-healing hydrogels with tunable mechanical properties have gained significant attention in tissue engineering and are desirable for organ regeneration, particularly for CTE [4,11,12].

**Figure 1:**
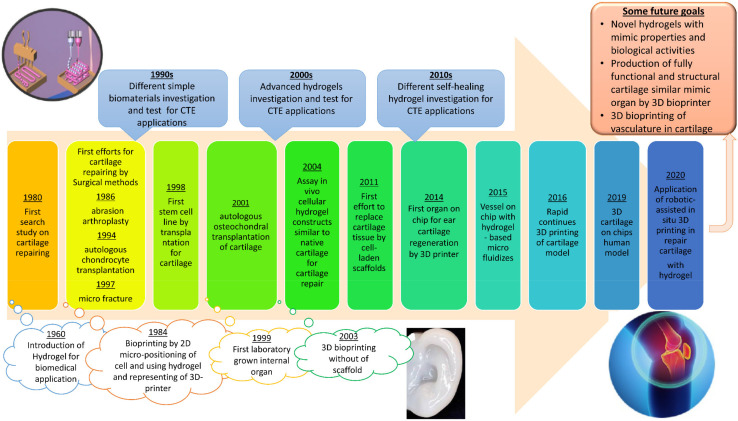
A timeline for the most important developments in the history of cartilage repairing with focus on hydrogel-based CTE. Reproduced with permission from Refs. [16,17].

The timeline of the major developments in CTE is presented in Figure 1. It started with the simple definition of hydrogel in 1960 and has reached the novel bioprinting for CTE using advanced hydrogels. Its focus on CTE for cartilage repairing also briefly addresses the development of hydrogels for CTE applications. Several review papers studied different natural and synthetic biopolymers and their properties, their recent advances including nanocomposites and interpenetrating networks, etc., fabrication of hydrogel scaffolds, and fillers utilized as hydrogel components for cartilage repairing [13,14,15,16]. Additionally, some papers considered self-healing hydrogels in tissue engineering. However, these articles did not provide an overall and comparative classification of the self-healing hydrogel for CTE application, which is essential from the material selection point of view. While several research groups have comprehensively reviewed self-healing materials, these publications have rarely focused on applying such systems in CTE. In the current manuscript, we concisely explained self-healing hydrogels in CTE. This review summarized the latest efforts for the preparation of hydrogels for cartilage-repairing applications, with a particular focus on advanced self-healing hydrogel. Therefore, the outstanding goal of the current review was to reinforce the importance of modification and improvement of the high-performance hydrogels in CTE.

## 2. Cartilage Structure

**Figure 2:**
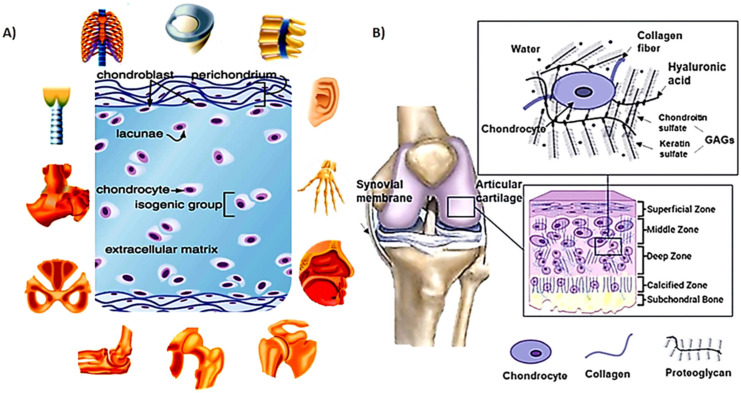
(**A**) Cartilage tissue component and its types in the human body; (**B**) main layer of hyaline cartilage tissue. Reproduced with permission from Refs. [3,22].

The main role of cartilage is to create a low-friction area inside synovial joints that provides the skeleton connections with load transmission capabilities during a range of motions in different activities [14]. The cartilage has a complex avascular and aneural structure. The superficial (external), the middle (central zone), the deep zone, and the calcified zone are the four basic layers of cartilage wherein their thickness depends on the ECM contents, structure, and chondrocyte status. Figure 2A,B shows components and various types and the four main zones of hyaline cartilage tissue [3].

In general, this complex texture is composed of water, various types of collagen, proteoglycans, and chondrocytes. The interaction of these components during cartilage formation leads to the formation of a robust tissue construct that can tolerate the incoming mechanical tensions and loads [3,18]. For example, articular cartilage tissue contains 70%–85% water and 60%–70% (of the dry weight of cartilage) collagen [19]. While collagen type II is the basis for articular cartilage and hyaline cartilage, collagen types I, III, V, VI, IX, XI-XII, and XIV also exist in the cartilage. Proteoglycans, as the next prevalent portion of cartilage, comprise around 30% of the dry weight of cartilage and are made of hyaluronic acid (HA) backbone with sulfated glycosaminoglycans (GAGs) branches [18]. The mentioned portions together are considered as ECM. Chondrocytes produce the ECM components; however, they organize 2% of the volume of mature cartilage [2].

An amorphous layer on the outer surface of the cartilage protects the cartilage surface and plays an essential role in lubricating the knee joint. This layer has approximately equal quantities of glycosaminoglycan (GAG), protein, and lipid [20]. Articular cartilage is also surrounded by a synovial fluid—made of water, hyaluronan, proteins, proteoglycans, and lipids—which acts as a lubricant to reduce friction between cartilage and meniscus surfaces [21].

### 2.1. Mechanical Properties of Cartilage

Articular cartilage made of a proteoglycan gel reinforced matrix by collagen fibers has the main task of load bearing and its distribution in the knee. Articular cartilage dis-plays properties such as high stiffness (fracture energy (toughness) ≥1000 J m^−2^ [20], stiff-ness ≥1 MPa [22]), and high tensile resistance (≥30 MPa) [21]. Tensile elongation of up to 140% limits its stretchability [23]. The most important factors in the design of the cartilage scaffolds are biocompatibility, biodegradability, enhanced cell differentiation, stability, suitable mechanical properties and porosity, cell adhesion, and accretion with the adjoining native cartilage [22,23]. Currently, design views for tough hydrogels have been focused on the effective damping of mechanical energy around the damaged area through the fracture of weak bonds. Tensile, compressive, permeability, and shear tests are the main techniques to determining cartilage mechanical properties and highly depend on test methods [24]. Additionally, the types of cartilage, strain value, depth from the articular surface, and maturation of cartilage tissue affect tensile, compressive modulus, and permeability of cartilage tissue. A wide range of 0.08–2.5 MPa is reported for the confined equilibrium compression modulus (for superficial and deep zones of cartilage, respectively). Additionally, the tensile modulus of the superficial layer in mature human cartilage is ~25 MPa, while it is 5.5 MPa in the middle zone and 4.5 MPa in the deep layer. The hydraulic permeability varies between 0.3 × 10^−17^ to 4.6 × 10^−15^ m^2^/ (Pa. s) in articular cartilage [21,24,25], 11.15 × 10^−16^–15.85 × 10^−16^ m^2^/(Pa. s) in the knee joint [26], and 0.9 × 10^−17^–2.9 × 10^−15^ m^2^/(Pa. s) in nose cartilage [27].

Mechanical properties including the equilibrium shear modulus, Poisson’s ratio, and aggregate modulus change notably for different joint positions. Additionally, these properties are dependent on the anisotropies existing within the cartilage, the sample area, and the sample orientation in test machines [21]. Some properties of cartilage, including Poisson’s ratio and Young’s modulus, do not depend on the applied test method and the alteration in these properties in different joint positions provides values within a particular range [21]. These typical values of the mechanical properties of cartilage are summarized in Table 1.

The biological properties are another main factor that depends on the mechanical properties of the tissue constructs. For example, the high loss angle and plastic deformation occur in highly porous scaffolds (pore size ≥250 μm), although the storage modulus and aggregate modulus reduce in such scaffolds. Mainly, small pore size (≤200 μm) causes lower aggregate modulus, propagation, and transition of nutrients and essential material; so, the suitable pore size and its propagation are essential for cell delivery and development of cartilage tissue [18]. Additionally, surface roughness and friction coefficient are two measurable tribological characteristics in both native and engineered articular cartilage. Native cartilage has a smooth and shiny surface with friction coefficients as low as 0.001 up to physiologically high pressures. However, human-made hydrogels are unable to match that of the native tissue [20,30].

In addition, different scaffold materials are identified with distinct chondrocytes and mechanical and biological properties and could be utilized for controlling the mechanical properties of the engineered cartilage [31]. For example, the mechanical properties of poly(glycolic acid) (PGA) scaffolds are higher than the one with similar geometry made of poly(lactic acid) (PLA). Additionally, synthesized polyglyconate (PG) increases in poly(glycolic acid) scaffolds, whereas the synthesis of collagen type II enhances in collagen-based scaffolds [29]. Using an IPN scaffold helped to minimize the surface roughness of the scaffold by approximately 80%.

### 2.2. Damage and Treatment of Cartilage

Articular cartilage defects are one of the major clinical challenges for orthopedic surgeons and typically begin due to trauma, unusual mechanical forces during an activity, aging, etc. Due to the limited ability of cartilage to self-regenerate, local lesions can lead to debilitating joint pain, functional impairment, and degenerative arthritis [24]. Articular cartilage incapability self-repair is attributed to its avascular nature of cartilage tissue, catabolism reflex to pathological inter-mediators, finite capability of progenitor cells migration, proliferation, and ECM production [32]. Nowadays, two main healing techniques are applied to treat cartilage injuries: surgical approaches and tissue engineering [33]. Depending on structural defects in the cartilage, an inevitable surgical intervention including osteotomy, autologous osteochondral graft transplantation, or total joint replacement is required. Chondroprogenitor cells from bone marrow or blood cannot easily reside within the injured area of the cartilage to repair it; therefore, the healing process begins within a few weeks. The tissue regenerated by the cartilage self-healing process is generally fibrotic and has poor mechanical and structural properties compared with the native healthy tissue [32,34]. The available surgical methods such as microfracture, mosaicplasty, transplantation of autologous chondrocyte transplantation and supportive matrix methods, and osteochondral allograft for healing of cartilage defects can alleviate pains and recover joint functions with great success [34]. However, cartilage regenerated by these methods does not recapitulate all the physiological specifications of natural cartilage and, thus, it does not last for a long period. For instance, the regenerated tissues usually contain collagen type I, which has undesirable chemical and mechanical properties [34].

Over the past two decades, tissue engineering has become one of the most popular methods to regenerate cartilage tissue and reconstruct it [16]. Two main procedures for the biofabrication process applied in CTE include cell-laden bio-inks for direct fabrication of structures and cell-free methods for scaffold constructions. However, in most cases, cartilage produced has a high fiber content and does not exhibit the ideal characteristics of hyaline cartilage because of the finite differentiation of stem cells. So, many research studies have been performed to improve mesenchymal cell-mediated osteochondral lesions and to increase the ability of stem cell differentiation, in order to create an organized chondral tissue with a cellular content that emulates ideal hyaline cartilage and ECM [2].

## 3. Cartilage Tissue Engineering

Since the 1990s, different biomaterials (natural and/or synthetic materials, chondrocytes, stem cells, growth factors) have been explored and examined in CTE for injection or scaffold implantation [35]. The main types of scaffolds are polymeric films, hydrogels, and fibrous scaffolds [36,37]. Among them, hydrogel scaffolds made of natural resources have become more popular due to their comparable structure and biological properties to native ECMs, which facilitate cell transplantation, proliferation, and differentiation [35,37].

The most important factors in the design of cartilage scaffolds are biocompatibility, biodegradability, cell differentiation and cartilage creation, stability, suitable mechanical properties and porosity, cell adhesion, and accretion with the adjoining native cartilage [16]. Because the cartilage mechanical properties may significantly change due to the age, joint donor site, and specimen orientation, various methods and procedures have been developed to improve the mechanical properties of an engineered cartilage. These include controlling the fiber geometry of the scaffolds, controlling the composition of ECM made by the chondrocytes, and the selection of different scaffold materials [21,36,38]. Scaffolds should have cell adhesion ligands, including epitopes for cells-surface interactions. This type of scaffolds can improve cell adhesion, cell differentiation, and integration into the surrounding native tissues. The ability to fill the irregular shape of the lesion and specific cell differentiation in damaged areas are other requirements for an ideal biomaterial scaffold used for delivering cells for cartilage healing [39]. Additionally, the mechanical properties of cartilage change with distance from the articular surface, maturation, degree of cartilage defect, and the capacity of compression and stress. Therefore, the desirable biomaterials for cartilage regeneration should be adjustable to mimic a range of the compression and tensile properties [40].

### 3.1. Hydrogels for Cartilage Healing

Hydrogels are 3D crosslinked hydrophilic networks of polymers or macromers that swell in an aqueous environment and create a platform for cells to proliferate and differentiate similar to a native ECM. The unique properties of hydrogels are mainly due to their high water content, specific structure, the ease of loading growth factors, and their mechanical properties [41]. Ideal biomaterials and specific production methods have main roles in improving desirable hydrogels that could be applied as CTE scaffolds [42].

**Figure 3:**
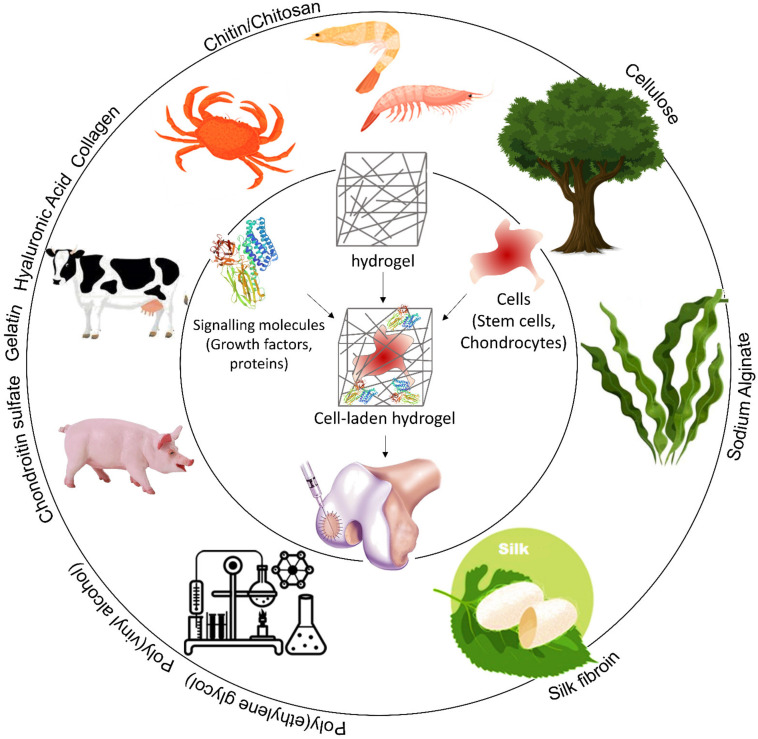
Schematic representation of different biopolymers used for CTE.

A wide range of natural and synthetic biopolymers [18,43] have been tested to produce hydrogels such as chitosan [44,45], collagen [46] or gelatin [47,48], alginate [49], hyaluronic acid [50,51], heparin [52,53], chondroitin sulfate [54], poly(ethylene glycol) (PEG) [55], and poly(vinyl alcohol) [56] (Figure 3). Table 2 summarizes the studies on the most popular biopolymers used to repair cartilage.

**Figure 4:**
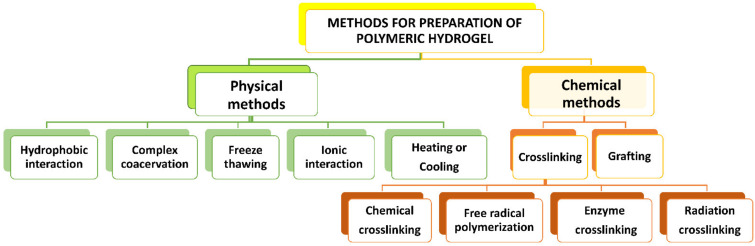
Various methods of hydrogel production.

Hydrogels could be produced by physical and/or chemical processes. A schematic diagram of various methods of hydrogel production is represented in Figure 4. Physically prepared hydrogels are created via relatively weak intermolecular forces, but chemical methods of hydrogels preparation are typically created by covalent crosslinking [13].

The viscoelasticity of hydrogels permits load transmission to chondrocytes [43]. Although different hydrogels produced by various procedures have been investigated in re-cent years, rarely have any integrated hydrogels been used in the regenerative medical profession. Hence, novel injectable hydrogels with improved physicochemical properties are required for cartilage regeneration in vivo [4,57].

### 3.2. Advanced Hydrogel for Cartilage Tissue Engineering

Hydrogels based on single-polymer networks usually illustrate very poor mechanical properties compared with native cartilage [94]. For example, alginate is utilized as a single-component hydrogel because of its properties such as biocompatibility and ionic crosslinking using calcium ions. However, it is almost bioinert and has limited cell interaction and adhesion [95].

For improving the hydrogel mechanical properties to attain those of native cartilage, more complex systems of multiple polymers have been recently used. These systems not only display higher mechanical properties than single polymeric networks, but also illustrate better integration with living tissue environments [96]. Recent advances in the hydrogel are categorized into four main groups: multi-material hydrogels, supramolecular hydrogels, nanocomposites hydrogels, and interpenetrating network (IPNs) hydrogels [97].

Multi-material hydrogels are one of the studied bioinks to dominate the restrictions of single-component hydrogels. In multi-material hydrogels, different single polymeric components are crosslinked together chemically to improve the final characteristics of the composite construction [98]. Supramolecular polymers are another currently under study. Supramolecular (host-guest) interactions happen physically between two or more chemical portions via non-covalent bonds [99,100]. In a supramolecular interaction, a guest segment of the polymer chains is connected to a host segment by non-covalent interactions all over the hydrogel network [101,102].

Due to the reversibility of these interactions, formed polymer networks illustrate self-healing properties. The most popular self-healing polymers that involve in guest-host interactions are based on cyclodextrins, cucurbits. Under high tensions or forces, these non-covalent interactions are reversibly broken for damping network energy. The reversible bonds also develop shear-thinning properties that facilitate their application in CTE [103,104]. Jeong et al. modified hyaluronic acid (HA)-based hydrogels by β-cyclodextrin. Resulted hydrogels demonstrated remarkable shear-thinning, cell viability, adhesive strength, and desirable mechanical properties for CTE applications [105]. Until now, several hydrogel substances have been employed for stem cell therapy. Salati et al. reviewed the sources and superior properties of the agarose-based bio-materials with/without various types of signaling molecules and their functions in the keeping of cartilage homeostasis [18].

Nanocomposite hydrogels have been studied to tailor the properties of hydrogels. The addition of nanoparticles to the hydrogel matrix can considerably change physical and chemical specifications of the scaffold, such as compressive strength, elastic modulus, storage modulus, and degradation rate under physiological conditions [106]. Depending on nanoparticles applied to the hydrogel network, interesting functions and properties such as bioactivity, controlled drug delivery, and electrical conductivity are induced to the hydrogel matrix [107]. However, despite numerous advantages of nanocomposite hydrogels, few studies have investigated their applications for cartilage.

Mechanically tough hydrogels with limited deformation under cyclic loads are highly desirable in cartilage tissue regeneration [108]. When subjected to cyclic stress, conventional hydrogel bonding can break due to the lack of mechanical integrity. To improve the mechanical properties of hydrogel-based scaffolds, Piluso et al. developed a 3D nanocomposite hydrogel composed of gelatin and starch nanocrystals (SNCs). The incorporation of SNCs within the hydrogel matrix led to an increase in the compressive modulus from 2.0 ± 0.1 kPa to 3.1 ± 0.1 kPa when the SNCs content increased from 0 to 0.5 wt%, compared with the hydrogel without SNCs [109]. Asadi et al. studied the role of various nanoparticles such as polymeric and silica nanoparticles in CTE [107,110]. They developed nanocomposite hydrogel scaffolds via combining gelatin and polycaprolactone–polyethylene glycol (PCL–PEG–PCL) nanoparticles which are loaded with transforming growth factor β1 (TGFβ1) and evaluated their potential as scaffolds for cartilage tissue engineering. Their results demonstrated a higher Young’s modulus of nanocomposite scaffold compared with the gelatin scaffold after the addition of PCL–PEG–PCL nanoparticles [110]. Bonhome-Espinosa et al. reported the fabrication of a novel magnetic 3D fibrin-agarose hydrogel using encapsulated magnetic nanoparticles and human native chondrocytes with the possibility of applying as articular cartilage tissues. The produced hydrogel showed excellent biocompatibility, viability, and proliferation in vitro [111]. The combination of nano-hydroxyapatite (n-HA) and magnetic nanoparticles (Fe_2_O_3_) with poly(vinyl alcohol) (PVA) could also produce a magnetic nanocomposite hydrogel with tensile strength of ~28.7 MPa [112]. In another study, Nejadnik et al. investigated the addition of calcium phosphate nanoparticles to bisphosphonate-functionalized hyaluronic acid for knee cartilage tissue engineering. The resulting hydrogel illustrated superior properties including self-healing [113]. The development of injectable nanocomposite hydrogels with mechanical properties comparable with bovine cartilage was reported by Schlichting and co-workers. They fabricated a photopolymerizable PEG-1000 and Pluronic F-127 hydrogels embedded with calcium phosphate nanocrystals via an in-situ mineralization technique. Their nanocomposite hydrogels had compressive and shear modulus of 0.64  ±  0.1 MPa and 1.5–2 GPa, respectively, slightly higher than those of bovine cartilage (0.35  ±  0.1 MPa and 0.7 GPa, respectively) [114].

Compared with multilateral composite hydrogels, interpenetrating networks (IPNs) are composed of independent polymer networks physically entangled to one another [115]. They are usually formed using different crosslinking methods and agents to solely crosslink one type of polymers within the network. Therefore, a network of independently crosslinked polymers are created that shows improved mechanical properties compared with their single-component network counterparts [116]. Generally, the primary polymeric network is made of flexible and elastic materials compared with the secondary network, which is stiffer and more brittle in relatively lower concentration [115,116]. Schipani et al. studied mechanically reinforced IPN hydrogels of alginate and gelatin methacryloyl (GelMA) reinforced by polycaprolactone (PCL) fibers. Motivated by the significant tension-compression nonlinearity of the collagen network in articular cartilage, they printed PCL networks to reinforce IPN hydrogels. This new composite hydrogel exhibited dynamic and equilibrium mechanical properties that approached or matched those of healthy articular cartilage [117]. Advanced hydrogels are summarized within the framework of different network types for cartilage tissue application in Table 3. Although a few research groups have reported the design of materials with predetermined properties and precise computational or mathematical models [118] to find their unique applications, the majority of researchers have simply combined new materials based on trial and error and characterized the properties of the final products [4]. These models provide us with useful tools to control hydrogel properties depending on the damaged cartilage area and its characteristics.

Stimuli-responsive hydrogels (SRHs) have gained great attention in drug delivery [119] and tissue engineering [120] due to their capability to undergo physical or chemical changes in response to external stimuli or small alterations in their environment. In contrast to the static hydrogel scaffolds, stimuli-responsive scaffolds have emerged as powerful platforms to dynamically respond to the cytocompatible stimuli, thus enabling on-demand manipulation of cell microenvironments. To induce such dynamic behaviors into the scaffolds, various physical (e.g., temperature [121,122], electrical or magnetic fields [123], wavelength or intensity of light [124,125,126], ultrasound [127]), chemical (e.g., pH [128,129], ionic strength [130], chemical triggers [131]), and biological (e.g., enzymes [132]) stimuli have been introduced. The extent of the responses to such triggers strongly depends on the nature and magnitude of a stimulus and the sensitivity of materials.

Temperature-responsive hydrogels that undergo physical sol-gel transitions can be easily implanted via minimally invasive operations without the need for external crosslinking agents [133]. However, the transition temperature, gelation time, and pH of polymer solutions should be precisely adjusted to meet clinical requirements and minimize detrimental effects on cell viability. N-isopropylacrylamides (NIPAAm), poloxamers, and different PEG-based polymers are common examples of temperature-responsive hydrogels [134]. Sá-Lima et al. explored the ability of poly(N-isopropyl acrylamide)-g-methylcellulose (PNIPAAm-g-MC) hydrogels (with lower critical solution temperature of ~32 °C) in supporting cell encapsulation and GAGs production, required for cartilage regeneration [135]. Park et al. introduced an injectable thermo-sensitive chitosan-pluronic hydrogel as a potential candidate for CTE [136]. This hydrogel demonstrated a transition temperature of ~25 °C and could support the proliferation of bovine chondrocytes and the synthesis of glycosaminoglycan for 28 days. Recently, Abbadessa et al. synthesized methacrylated pHPMA-lac-PEG hydrogels (a thermo-responsive triblock copolymer) for cartilage 3D bioprinting [137]. It was found that the incorporation of polysaccharides (methacrylated chondroitin sulfate (CS-MA) or methacrylated hyaluronic acid (HA-MA)) could improve the stability and printability as well as the mechanical properties of the hydrogel-based constructs.

The pH-responsive hydrogels contain acidic or basic functional groups with proton exchange capability, depending on the pH of the surrounding environment. Strehin et al. synthesized a pH-responsive chondroitin sulfate (CS)—PEG adhesive hydrogel with potential applications in regenerative medicine, including cartilage repair [138]. It was found that changes in the initial pH of the precursor solutions could impact the stiffness, swelling properties, and kinetics of gelation of the final hydrogel products. In another study, Halacheva et al. developed pH-sensitive hydrogels from poly(methacrylic acid)-containing crosslinked particles with high porosity, elasticity, and ductility [139]. The enhanced mechanical properties of the produced hydrogels made them a suitable candidate for regenerative medicine. Sá-Lima et al. designed pH-sensitive hydrogels based on chitosan-β-glycerophosphate-starch with the ability to induce chondrogenic differentiation of adipose-derived stromal cells (ADSC) for CTE [140]. Despite considerable progress in the development of pH-responsive hydrogels for cartilage regeneration, it is still difficult to clinically predict the pH at the diseased site, which may cause undesired tissue response.

Light-sensitive hydrogels can also be applied for CTE. Levett et al. prepared photo-crosslinkable hydrogels based on gelatin methacrylamide that enhanced chondrogenic differentiation and improved mechanical properties of the regenerated cartilage [141]. Giammanco et al. developed photo-responsive hydrogels composed of alginate–acrylamide hybrid gels and ferric ions [142]. The physicochemical properties of these hydrogels could be modulated using visible light irradiation. While better spatial and temporal control over precursor gelation can be achieved via photo-crosslinking processes, their in vivo applications can be restricted due to the potential toxicity of photo-initiators at an elevated temperature over a prolonged irradiation period.

Chemical bonds formation and cleavage by enzymes can also be utilized for hydrogels formation. Skaalure et al. synthesized aggrecanase-sensitive hydrogels based on photo-clickable thiol-ene PEG that contains a CRDTEGE-ARGSVIDRC peptide, derived from the aggrecanase-cleavable site in aggrecan [132]. The bovine chondrocytes encapsulated within this hydrogel produced a connected matrix rich in aggrecan and collagen II, but not collagens I and X. In contrast, the matrix deposition in the non-degradable hydrogels (i.e., control groups) consist of aggrecan and collagens I, II, and X, indicative of hypertrophic cartilage. Jin et al. prepared injectable chitosan-graft-glycolic acid (GA) and phloretic acid (PA) (CH-GA/PA) hydrogels enzymatically crosslinked via horseradish peroxidase (HRP) and hydrogen peroxide (H_2_O_2_) [143]. They also synthesized injectable hydrogels using hyaluronic acid-dextran-tyramine conjugates with potential applications for CTE [144]. The hydrogels were formed via enzymatic crosslinking of tyramine residues in the presence of HRP. Nevertheless, the high concentration of H_2_O_2_ during injection may cause cytotoxicity in these hydrogels in vivo [145]. For the development of smart scaffolds that can respond to various stimuli, we direct readers to an excellent published review in the literature [119].

## 4. Self-Healing Hydrogel in Cartilage Tissue Engineering

Recently, self-healing soft systems with large deformation capabilities have been developed using multiple crosslinking mechanisms. These materials have attracted significant attention due to their extensive applications in electronics, coatings, and biomedical prosthetics [12]. Self-healing enables materials to repair themselves and restore their morphology and mechanical properties after defects. This ability not only maintains the longevity of a system, but also enhances the mechanical stability and prevents sudden or permanent failure of such materials in sensitive applications [165].

**Figure 5:**
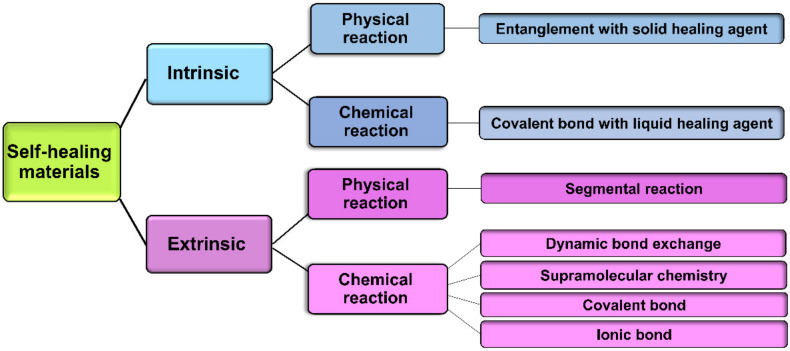
Schematic classification of self-healing schemes.

One of the basic self-healing techniques for the repair of polymer network defects is the increase of temperature. Thermoplastics, i.e., polymers that can be melted and re-cast almost indefinitely, benefit from a simple self-healing mechanism activated upon heating to a temperature above their melting points [166]. However, they usually show low stiffness and thermal stability which limit their applications where mechanically robust structures are needed. Therefore, self-healing research has mainly focused on thermosetting polymers, i.e., polymers that are irreversibly hardened by heat [167]. In thermosets, however, the intrinsic chain mobility within the polymer network is slow or negligible compared with thermoplastics. While heating is a simple method of self-healing, the restrictions excreted by the physicochemical properties of new materials have made an inevitable need for the development of new techniques to accelerate the self-healing process. Self-healing approaches can be classified into (i) intrinsic healing, due to an inherent ability of materials to self-heal, triggered either by a damage or in combination with an external stimulus and (ii) extrinsic healing, based on the release of the healing agents (e.g., liquid agents such as catalysts, monomers, hardeners containing microcapsules and hollow fibers embedment), pre-embedded into the (polymeric) matrix, upon damage [168]. In general, both intrinsic and extrinsic processes can be accomplished via physical self-healing by chain entanglements and chemical self-healing by the recovery of chemical bonds (e.g., hydrogen bonds, covalent bonds, etc.) [169], shown in Figure 5.

**Figure 6:**
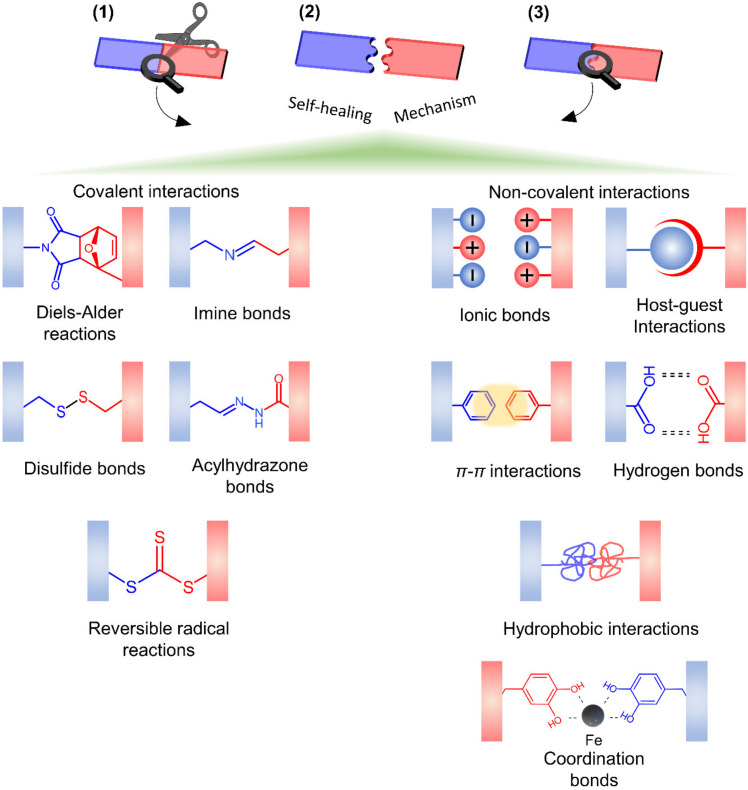
Main self-healing mechanisms and polymer behavior in hydrogels. (**1**). Damage occurrence, (**2**). Self-healing process, and (**3**). Healed hydrogel.

Self-healing in hydrogels are prepared via dynamic covalent reactions (chemical crosslinking) and/or non-covalent reactions (physical crosslinking) [23] shown in Figure 6. In covalent reactions, the re-use of polymerization conditions or the utilization of an external stimulus (e.g., heat [148], pH [170], UV, visible light [171]) is necessary for the completion of the healing process. In contrast, autonomous self-healing generally occurs in materials without using an external stimulus and leads to partial or full recovery of their physicochemical characteristics (e.g., mechanical properties). Non-covalent interactions commonly employ an individual or a combination of bonding mechanisms such as ionic bonding [172], hydrogen-bonding [173,174], supramolecular interactions [101], hydrophobic bonding [175], and molecular diffusion and chain entanglement [169].

### 4.1. Materials

Self-healing hydrogels made from either natural or synthetic polymers can be obtained by incorporating functional groups mentioned above in the polymer backbones via various non-hazardous and non-toxic chemical modifications. Natural hydrogels used for self-healing hydrogels include plant-derived hydrogels (e.g., polysaccharide-based alginate, carboxymethyl cellulose, cellulose, and agarose) and animal-derived hydrogels (hyaluronic acid, gelatin, chitosan, collagen, and fibrin). Synthetic hydrogels are based on polymers such as poly (ethylene glycol), poly (acrylic acid), poly (vinyl alcohol), and polyacrylamide [168,176]. It is possible to use a combination of a synthetic and natural polymer to produce novel hydrogels with remarkable positive properties of both components [168]. Roh et al. combined polysaccharide-based hydrogels with alginate to reinforce self-healing and properties such as stability, viscoelasticity, and printability by dual crosslinking for CTE application [177]. Wang et al. produced a dual responsive hydrogel based on oxidized sodium alginate (OSA) and hydrazide-modified poly(ethyleneglycol) (PEG-DTP) with injectability and self-healing properties. OSA has weak properties at low PH. However, PEG-DTP efficiently enhanced the flexibility, self-healing, mechanical properties, and hydrophilicity of OSA due to the reversibility of its dynamic acylhydrazone connections. The resulting hydrogels illustrated self-healing of approximately 100% after damage [178]. In another study, Yu and coworkers introduced a multifunctional hydrogel of hyaluronic acid, furylamine (furan), and adipic dihydrazide. Combination of Diels-Alder click reaction and acylhydrazone bond enhanced integrity and mechanical performance of this hydrogel in a biological environment, although the dynamic covalent bond of acylhydrazone created an excellent autonomous self-healing property and cell-adhesion for CTE applications [179]. However, other self-healing hydrogels based on peptides, mussel-inspired proteins, conductive polymers, and zwitterionic polymers have also obtained attention in recent years; however, they are not suitable for cartilage tissue engineering [167,169].

### 4.2. Mechanisms of Self-Healing

Generally, intrinsic self-healing hydrogels are preferred in CTE applications due to their superior advantages in the restoration of their functions without adding new chemicals. Re-crosslinking damaged scaffolds via chemical reactions of different functional groups or physical interactions is the main objective in all intrinsic self-healing processes (these mechanisms have been discussed in detail elsewhere [180]). The intrinsic self-healing is dependent on reversible crosslinking. The type and strength of bonds (used as crosslinkers) define the degree of self-healing, durability, and the mechanical properties of repaired hydrogels. Thus, they are the main factors in designing hydrogels with specific applications. For example, hydrogels made of physical crosslinking via hydrogen bonds are mechanically weaker than covalently crosslinked hydrogels of the same materials [180,181,182]. The bonding energy of hydrogen bonds is typically in the range of 5 to 30 kJ mol^−1^, around 10 times weaker than that of covalent bonds (≈345 kJ mol^−1^ for C-C bonds). The energy of the hydrogen bonds mostly depends on the negative charge of acceptor atoms (i.e., O, N, F) and, therefore, it varies significantly with the electronegativity of acceptor atoms and pH of the solution in which the interactions occur. The strongest hydrogen bonds are associated with hydroxyl (-OH) or amide (-NH) groups while the weakest are those that incorporate fluorine.

**Figure 7:**
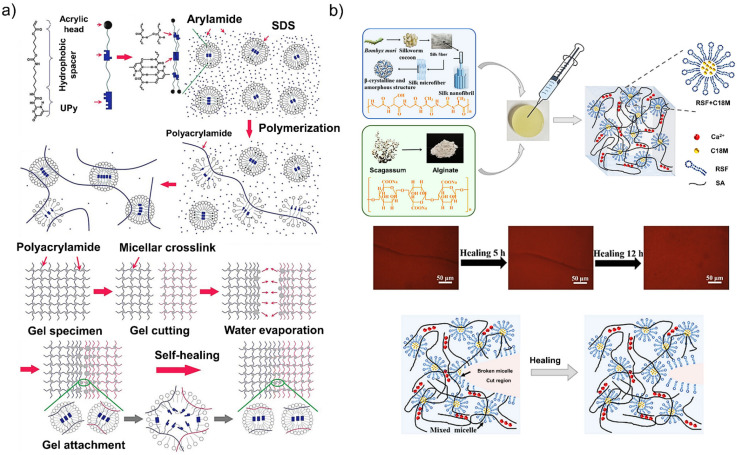
(**a**) Schematic illustration of the micellar copolymerization of the UPyHCBA and acrylamide. The self-healing mechanism (i.e., hydrophobic interactions) of the micellar copolymerization hydrogels [183]. (**b**) Schematic illustration of the preparation process of silk fibroin-based hydrophobic-association hydrogels; optical images of the self-healing process of hydrogels over time; hydrogel region before and after healing. Reproduced with permission from Ref. [184].

Hydrophobic interactions play an essential role in biological systems for shape changing of proteins in water-rich environments and membrane formation. These interactions are slightly stronger than hydrogen bonds and can be easily modulated through altering the shape and the balance of hydrophobic and hydrophilic moieties in a system [183]. In intrinsic self-healing hydrogels, the presence of hydrophobic interactions leads to the re-arrangement of hydrophobic blocks to reduce or eliminate contacts with water molecules. Jeon et al. introduced novel hierarchical systems of non-covalent crosslinks with excellent stretchability and damage recovery created by incorporating amphiphilic polymers (UPyHCBA with an acrylic head, a hydrophobic alkyl spacer, and a 2-ureido-4-pyrimidone (UPy) tail) and surfactants (sodium dodecyl sulfate) into polyacrylamide hydrogels (Figure 7a) [105]. The obtained hydrogels were able to stretch ~100 times their initial length and to intrinsically self-heal within ~30 s. Using reversible hydrophobic interactions, Meng et al. fabricated silk fibroin-based hydrophobic-association hydrogels incorporated into an alginate ionic network (Figure 7b) [184]. This new system demonstrated excellent biocompatibility, mechanical properties, and intrinsic self-healing behavior without applying external energy at room temperature.

**Figure 8:**
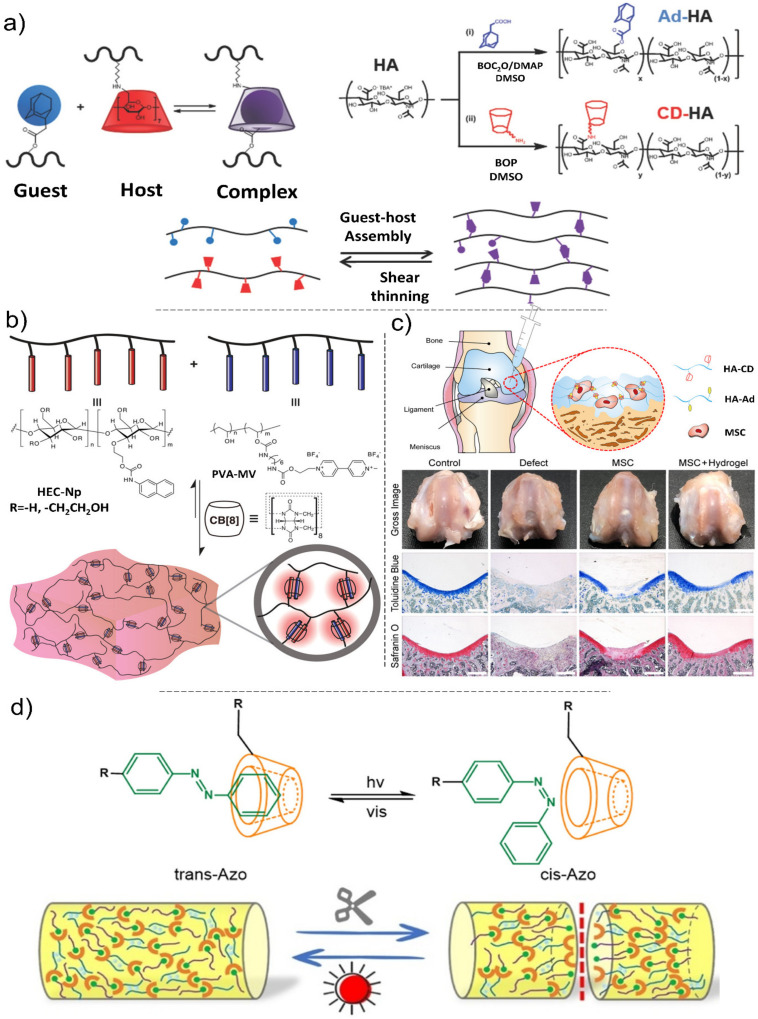
(**a**) Guest–host hydrogel formation based on the complexation of adamantane and β-cyclodextrin and corresponding synthesis processes. Schematic representation of hydrogel formation via guest–host interactions. Reproduced with permission from Ref. [193]. (**b**) Schematic illustration of a supramolecular hydrogel based on host–guest complexation with cucurit[8]uril (CB[8]). Reproduced with permission from Ref. [191]. (**c**) Left: Schematic illustration for injectable supramolecular hydrogels encapsulating MSCs for cartilage tissue regeneration. Right: optical images and histological analysis of regenerated cartilage tissues after treating with hydrogels and MSCs. Reproduced with permission from Ref. [105]. (**d**) The host-guest interactions between Azo derivatives and CD derivatives in the presence and absence of light and the schematic illustration of the alginate-based cyclodextrin/azo-polyacrylamide composite self-healing process. Reproduced with permission from Ref. [196].

Due to the limited research on the suitable dynamic chain mobility of supramolecular and component interactions, the production of self-healing materials with versatile mechanical properties still remains a challenge [185], impeding their real-world applications that require mechanical integrity. Recent advances in supramolecular chemistry have accelerated the development of an increasing number of biologically inspired hydrogels [186,187]. Biopolymers physically crosslinked via host–guest interactions in supramolecular hydrogels have shown great potential for the development of minimally invasive therapeutics [188]. Most of these hydrogels demonstrate shear-thinning behavior under shear stress and recovery (i.e., self-healing) when the shear force is removed. However, these systems generally rely on nonspecific interactions, leading to protracted recovery times (from minutes to hours) following the shear stress removal [189]. This limits the efficacy of injectable hydrogels in the immobilization of material components or encapsulated cargos (e.g., cells, growth factors, etc.) at a target site. To overcome this problem, host−guest interactions based on non-covalent bonding of a macrocyclic host (e.g., cyclodextrin (CD)) and a complementary guest molecule (e.g., adamantane) have been introduced (Figure 8a,b) [190,191,192,193]. Generally, host−guest hydrogels are mechanically weak [194,195]. This issue significantly limits their widespread applications in tissue engineering, particularly in load-bearing tissues such as cartilage. Recently, Jeong and co-workers reported injectable hydrogels based on β-cyclodextrin-modified hyaluronate and adamantane-modified HA, encapsulating mesenchymal stem cells (MSCs) for CTE applications. These hydrogels demonstrated remarkable mechanical characteristics including shear-thinning and self-healing with high cell viability [105]. The therapeutic efficacy of the HA hydrogels/MSCs for cartilage tissue regeneration was evaluated in vivo (Figure 8c), where the hydrogels/MSCs confirmed better macroscopic neocartilage formation covering the entire defect area compared with control groups. He et al. introduced a highly stretchable and tough alginate-based cyclodextrin/azo-polyacrylamide composite with self-healing properties via light irradiation [196] (Figure 8d). The azobenzene group used in the chemical structure of these hydrogels is a light-responsive group that experiences a reversible transformation between a cis structure (under light irradiation) and a trans structure (in the absence of light). Therefore, host-guest interactions between Azo derivatives and CD derivatives under the dark condition yielded hydrogels with the tensile strength of 0.06 MPa at 1819% strain, where the presence of calcium ions crosslinking alginate chains increased the tensile strength but reduced the elongation of hydrogels. While many self-healing hydrogel platforms currently exist, Table 4 highlights the most promising systems for cartilage tissue engineering.

Despite extensive research on improving the mechanical strength of self-healing hydrogels, these systems still encounter serious challenges within the vibrant and mechanically demanding environment. To address this issue, scientists have developed hydrogels crosslinked via multiple dynamic as well as covalent bonds. While the number of research articles reporting self-healing hydrogels with multiple crosslinks for cartilage tissue engineering is limited, Table 5 summarizes the recent studies with potential applications in CTE. Qin et al. studied using reversible noncovalent bonds along with permanent covalent crosslinks to increase the mechanical strength of hydrogel to 34.0 MPa [218]. Yanagisawa and coworkers fabricated noncovalently crosslinked hydrogels with a low molecular weight and tensile strength of almost 26.5 MPa [219]. Ding et al. fabricated cross-linked hydrogel via both ionic- and hydrogen-bonds by applying acrylic acid and acrylamide, xanthan gum, and guar gum, which demonstrated excellent mechanical characteristics and moderate water content for use in the CTE [205].

## 5. Fabrication Methods

The precise fabrication of bio-scaffolds is among the main aims of tissue engineering research. Traditional scaffold fabrication techniques such as foam processing, solution casting, and freeze-drying have limited control on the chemistry, macrostructure, and porosity of final products. Electrospinning and 3D bioprinting are two advanced manufacturing technologies for making desirable tissue engineering scaffolds [235,236]. Scaffolds prepared using these two techniques are hollow matrices that support cell structures and improve cell adhesion and proliferation due to their highly porous geometry which facilitate the transport of oxygen, nutrients, and biological wastes. The most popular fabricating methods are listed in Table 6.

**Figure 9:**
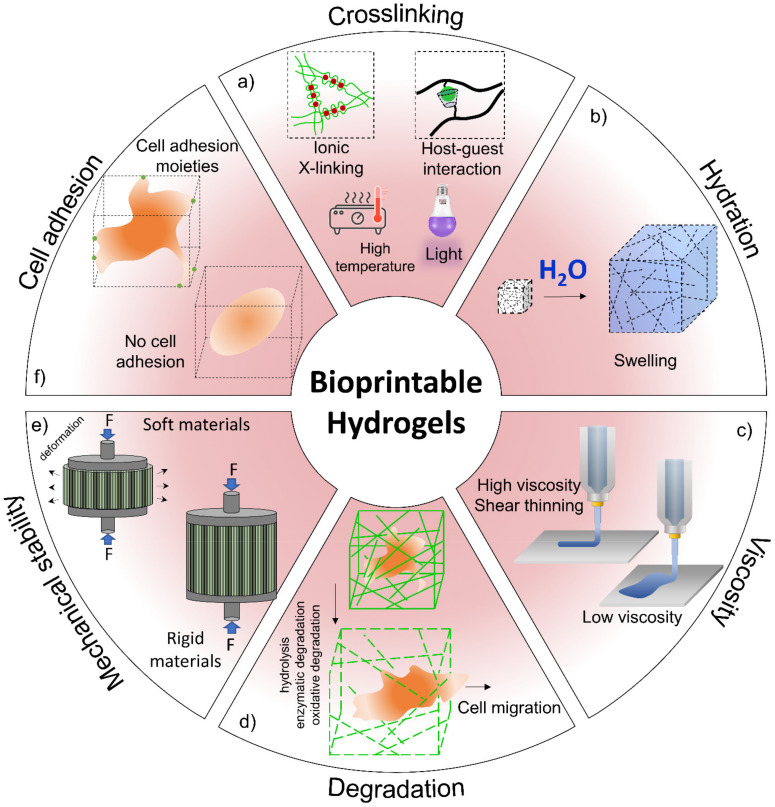
Hydrogel properties in bioprinting. (**a**) The crosslinking mechanism is related to polymer types that impact cell viability and structure properties. (**b**) Hydration of a hydrogel system facilitates nutrients and wastes transport within a printed structure. (**c**) Cell adhesion that supports cell proliferation and migration can be controlled by changing the polymer type. (**d**) Degradation mechanisms can influence cellular migration. (**e**) The durability of 3D printed structures is essential to mimic native tissue biomechanical properties and to retain the shape of constructs during cellular growth. (**f**) Viscous solutions can suspend and protect cells from shear stress inside an extrusion nozzle and reduce flowability of hydrogels after printing (low viscosity solutions can avoid clogging; however, cell settling may occur).

3D printing, a growing additive manufacturing technology for fabricating precise 3D structures, is currently widely used to increase the applicability and functions of cell-laden scaffolds. During the recent decay, tissue engineering has shown promising results for treatment of osteoarticular damage and has provided a suitable alternative to current therapies using 3D bioprinting methods in the clinical environment such as the use of various biomaterial scaffolds, allogeneic and autologous of chondrocytes bases, chondroprogenitor cells and growth factors, and mixtures of them [235]. The bioprinting process is based on the combination of various living cell-laden biomaterials referred to as bioinks [246,247]. The physicochemical properties of bioinks are very important to produce functionally live tissues such as cartilage. Thus, bioinks should have biological properties, biodegradability, and printability. Generally, hydrogels are a suitable candidate for bioinks preparation [17]. Figure 9 schematically presents the most important properties of bioinks and their effects on bioprinted constructs. With regards to cartilage regeneration, the hydrogel-based scaffolds are the primary biomaterials applied due to their bioadhesion and compatibility with the surrounding cartilage tissue environment. The physicochemical properties such as swelling ratio, surface tension, gelation time, and rheological parameters are the main factors affecting the printability of a hydrogel solution [17,236]. Roseti et al. reviewed the recent advances in bioprinting 3D scaffolds embedded with stem cells for CTE [248]. Additionally, Semba et al. introduced state-of-the-art 3D bioprinting techniques in cartilage and bone design for orthopedic applications [246]. Sadeghianmaryan et al. investigated the printability of chitosan scaffolds. They studied the effect of methods of drying, concentration, and crosslinking density on scaffold properties. They exhibited that the drying method is a critical character in the mechanical and biological performance of chitosan scaffolds. Additionally, smaller pore sizes and higher elastic modulus occur in higher crosslinking density at chitosan concentration of 10% [44]. Until now, many approaches have been reported for the production of proper bioinks, for the prediction of mechanical properties of a hydrogel structure after bioprinting, type of materials and additives, cell density, and material–cell interaction [249].

## 6. Conclusions and Perspective Remarks

Over the past decades, self-healing of damaged organs (due to trauma or degenerative pathology) in biological systems inspired researchers to develop new biomaterials able to mimic natural organs’ ECMs. Among these materials, hydrogels are attractive for clinical applications because of their high-water content and physicochemical properties, like what are found in native human tissues. Currently, with the advances of synthetic methods, a range of self-healing hydrogels has been expanded, introducing a new class of premium materials for specific applications in cartilage and bone repairing. However, current self-healing biomaterials are considerably suffering from weak and inadequate physicochemical and spatiotemporal properties and high production costs. Although various reversible bonding strategies are currently available for the development of new self-healing hydrogels, they do not meet all the specifications (e.g., high toughness and excellent elasticity, rapid self-healing, excellent integration with surrounding cartilage tissue, sufficient nutrition transportation, drugs and growth factors delivery, and printability) required for CTE.

On the other hand, clinical applications of new biomaterials can be limited by the cost and difficulty of passing safety and regulatory processes. FDA approval can pose a significant challenge to biomaterial-based therapies as new biomaterials need to meet FDA standards. Materials other than those already approved for use in humans have extensive requirements in quality control and safety. Therefore, when combining cells and materials, considerable animal and clinical testing is required, which comes with high costs and lengthy development timelines [250,251].

The creation of multi-functional self-healing hydrogels with multiple covalent/non-covalent bonds can significantly impact the future of CTE. Designing new materials using mathematical modeling and simulation methods offers interesting and cost-effective opportunities for generating new hydrogels mimicking the native tissue microenvironment. Modifications of currently available models would be another future direction to precisely predict the mechanical properties of hydrogels used for cartilage tissue regeneration [244,252,253].

Undoubtedly, one of the most promising future trends in the development of hydrogels is the combination of advanced hydrogels (nanomaterials, supramolecular, multi-materials, and IPNs) to make new composites with superior properties compared with every individual component. Combining high-performance hydrogels with the novel structure design, biological activity, and superior properties such as self-healing is a promising approach to repair cartilage defects. However, the lack of control over the structure of newly developed tissues is another challenge that can be addressed via multi-component 3D bioprinting technologies benefiting from higher resolution and faster printing speeds. The combination biofabrication methods are a relatively new approach in fabrication of bio-mimicking, heterogeneous, and complex tissue structures. Organ-on-a-chip, as an emerging technology that combines cell biology, engineering techniques, and biomaterials, can be utilized to simulate organs’ microenvironments on a microfluidic chip. These organ models recapitulating the main features of human physiopathology are highly desired to investigate new materials in terms of cell-tissue interfaces and metabolic performance. The combination of organ-on-a-chip and 3D bioprinting can provide even more realistic osteoarthritis models for testing new therapies. The opportunities for combination of approaches are tremendous and should motivate the field to push past technical and regulatory barriers, especially with the growing interest in personalized therapeutic approaches.

## Figures and Tables

**Table 1:** Mechanical and biological properties of natural articular cartilage.

Mechanical Property	Value	Test Method	Ref.
Aggregate modulus (MPa)	0.10–2.1	Confined compression	[22]
Hydraulic permeability (m^2^/Pa.s)	10^−16^–10^−15^	Unconfined compression, indentation	[22]
Compressive Young’s modulus (MPa)	0.23–0.85	Unconfined compression	[22]
Poisson’s ratio	0.05–0.30	Unconfined compression	[22]
Tensile equilibrium modulus (MPa)	5.0–12.0	Tensile stress relaxation	[22]
Tensile Young’s modulus (MPa)	5.0–25.0	Tensile constant strain rate	[22]
Tensile strength (MPa)	0.7–25.0	Equilibrium shear	[22]
Equilibrium shear modulus (MPa)	0.05–0.40	Equilibrium shear	[22]
Complex shear modulus (MPa)	0.2–2.5	Dynamic shear	[22]
Shear loss angle (°)	10–15	Dynamic shear	[22]
**Biological property**	**Value**		**Ref.**
Initial cell seeding	≥63 million cells/mL		[28]
Osmolality	Physiological osmolality		[29]
Extracellular pH	7–8		[29]
Pore size	2.5–6.5 nm		[29]
Growth factors	PDGF, TGF-β, FGF, BMP, IGF		[29]
Mechanical loading (dynamic compression)	2–10% strain or 0.5–1.0 MPa at physiological frequency 0.01 to 1.0 Hz		[29]

**Table 2:** Summary of the last 10 years studies on cartilage regeneration via biopolymers.

Main Base	Main Materials	Advantages	Highlighted Achievements	Disadvantages	Ref.
Chitosan	Chitosan, kartogenin	Increased mechanical properties, excellent biocompatibility, biodegradability, and cell adhesion	Significant statistical models to predict the properties	Immunogenic	[58]
	Gellan gum (GG)/nanoparticles/graphene oxide/hydroxyethyl cellulose/dialdehyde starch/ poly (vinyl alcohol)/gelatin/ hyaluronic acid		Controllable properties, degradation rate, and pore size		[43,59,60]
	Chitosan, Pyrrole		Good thermo-sensitive gelation	High gelation time, swelling, and degradation time	[61]
	Chitosan, PLA, calcium phosphate, hydroxyapatite		-	Bioinert	[62]
Collagen/gelatin	hyaluronic acid/dialdehyde micro fibrillated cellulose (DAMFC)/transglutaminase enzyme	Biosafe, excellent mechanical and biochemical properties, biocompatibility, and cell viability, low cost, biodegradable, ECM production of cartilage	-	Immunogenic	[63,64,65,66,67,68]
	Riboflavin, collagen, hyaluronic acid		-	Delayed enzyme-triggered degradation time	[69]
	Gelatin, graft-poly(N-isopropyl acrylamide)		Low water/oil interfacial tensions, thermo-responsive		[70]
	Alginate, borax		Reduced inflammatory effect		[71]
Hyaluronic acid	Alginate/cellulose nanocrystals, adipic acid dihydrazide/fibrin/ lysine methyl ester/divinyl sulfone, functionalized inulin	Bioprintable, biocompatible, good proliferation, stable, enhanced cell adhesion, proliferation, and differentiation	-	Weak mechanical integrity, fast degradation in vivo	[72,73,74,75,76,77,78]
	Polydactyly chondrocytes, heparin/fibrin		Cartilage-like matrix		[79]
	Trans glutaminase crosslinked hyaluronan		Excellent mitogen chondrification, superior adhesion to native cartilage		[80]
	PEG, chondrocytes		Superior mechanical properties, improved metabolic viability	Fast degradation	[81]
Fibrin	ECM microparticle, alginate microbeads/PEG, human amniotic fluid-derived stem cells	Stable, biocompatible, injectable	-		[82,83]
Alginate	Gelatin, Hydroxyapatite, protein (BSA),Alginate, Fibrinogen	Tunable mechanical properties similar to native cartilage, excellent osteochondral regeneration and proliferation, 3D printable, excellent cell adhesion and biocompatibility	Interconnected mesh structure, great flexibility and degradation	Slow and unpredictable degradation in vivo	[84]
	Hydroxyapatite (HAP) complex		-		[85]
	Bone marrow-derived mesenchymal stem cells/polymethacrylate hybrid, collagen type I/hyaluronic acid, elastin-like protein (ELP)		-		[86,87]
	-		Excellent viscoelasticity		[88]
	Gelatin		High degradation		[89]
Elastin	Poly(N-isopropylacrylamide- co-polylactide-2 hydroxyethyl methacrylate-co-oligo (ethylene glycol) monomethyl ether methacrylate (PNPHO)	Biocompatible, proper mechanical properties, good structural stable, cell proliferation, injectable	-	Difficult to integrate with surrounding tissue, Immunogenic	[90]
	Silk		Cell interactions		[91]
Chondroitin sulfate	Pullulan/poly (N-isopropylacrylamide) (NIPAAm)	Biocompatible, cytocompatible, increased cell proliferation, mechanically stable, improved cartilaginous ECM deposition, good mechanical properties, injectable	Self-healing	Immunogenic	[92]
			No cytotoxicity		[93]

**Table 3:** Summary of advanced hydrogel in cartilage tissue engineering.

Advanced Hydrogel Type	Main Materials	Advantages	Disadvantages	Ref.
Multi materials	Chondrocyte-laden GelMA, PCL	Porous structure, cell proliferation, excellent mechanical and thermo-reversible properties, printable	Long-time UV exposure and low cell viability	[146]
	PCL, Pluronic F-127	Biocompatible, biodegradable, finite antigenicity	Immune response and therapeutic efficacy have not determined	[147]
	Poly(vinyl alcohol), poly(ε-caprolactone), Gelatin methacrylamide/Gellan gum, Alginate	Great differentiation, ability to produce complex structure and support components	Low shape fidelity	[98]
IPN	Polycaprolactone, Poly (acrylic acid), Cellulosic nano-whisker, Acrylic-urethane cross-linker	Improved the mechanical properties, water absorption of about 30%, excellent hydrophilic property	Need to optimization of physicochemical surface conditions for cell adhesion and proliferation	[148]
	Carboxymethyl dextran, Amino dextran	Excellent mechanical stable, adhesion, and spreading behavior of fibroblast cells, biodegradable and biocompatible	Immune responses have not been determined	[149]
	Hydroxyapatite particles, Alginate	Proper osteochondral healing, suitable compressive modulus and swelling property, high porosity, uniform pores	Using of poor supramolecular gelation agent	[150]
	Conjugated dextran with 2-naphthylacetic, HA, β-cyclodextrin	Excellent resilience, good biocompatibility	N/A	[151]
	Gelatin, Alginate polyacrylamide	Enhanced mechanical properties, excellent cell proliferation, finite cytotoxicity, chondrogenic gene expression, and structural stability, great porosity in long-term	Uncontrollable porosity, Formation of a thin superficial layer that does not allow cell penetration	[152]
	Ethylene diamine-functionalized HA, Divinyl sulfone-inulin	Biodegradable, FDA-approved, good mechanical properties	Low cell viability	[78]
	Low-molecular-weight PVA, High molecular weight HA	Biocompatible, excellent swelling properties and cell viability	Fast gelation in room temperature	[153]
	Poly(ethylene glycol), Low-molecular weight HA	Excellent solubility in GAG deposition during structure maturation, support of collagen biosynthesis	Low enzyme degradation	[154]
	Methacrylated HA, Fibrin	Biocompatible, Support of differentiation	Unstable and unsuitable mechanical properties in low concentration	[155]
	Methacrylated chondroitin sulfate, Agarose-poly(ethylene glycol) diacrylate	Enhanced collagen biosynthesis and GAGs in the cell-matrix, low cost	Low cell viability	[156]
Supramolecular	Adamantane-functionalized HA, monoacrylated β-cyclodextrin	Great drying and re-swelling without changes in water content or shape, excellent collagen deposition, suitable biophysical properties, rapid stress relaxation, self-healing	N/A	[157]
	Cucurbituril, diaminohexane	Controlled dexamethasone release, enhanced cell proliferation, GAG synthesis, and chondrogenic gene expression, in vivo neocartilage production	N/A	[158]
Nanomaterials	Alginate, Poly(acrylamide) hydrogel, poly(lactide-co-glycolide) (PLGA) nanoparticles	Great viscoelasticity, biodegradable, biocompatible and protein absorber, excellent cell proliferation and mechanical strength, stable	N/A	[159]
	Poly(vinyl alcohol), Graphene oxide	Great bio-mechanical and bio-friction properties, excellent shear-thinning, printability, and printing accuracy, proper compressive and tribological properties	Unsuitable pore size	[160]
	Nano hydroxyapatite, Poly(vinyl alcohol), Poly(lactic-co-glycolic acid)	Biocompatible, practicable, excellent mechanical properties, sensitive to compressive stress, suitable chondrocyte adhesion and proliferation	N/A	[161]
	Poly(vinyl alcohol), Nano-hydroxyapatite, magnetic Nanoparticles (Fe_2_O_3_)	Proper mechanical properties, great mesenchymal stem cells growth	Variable crystallinity	[112]
	Hydroxypropyl methylcellulose, Laponites	Excellent mechanical properties, oxygen diffusion, and cell expression	Some toxicity, decreased cell density	[162]
	PEG, Laponite particles	Good elastic modulus, biocompatible, excellent mechanical properties	Low cell viability	[163]
	Silk fibers, Chitosan/Glycerophosphate	Excellent mechanical properties, GAG, and collagen type II expression	Unsuitable biological properties, toxic gelation agent	[164]

**Table 4:** Summary of intrinsic mechanisms of self-healing hydrogels in cartilage tissue engineering.

Mechanism Type	Materials	Self- Healing Conditions	Time of Healing	Properties	Main Reactions	Healing Efficiency	Ref.
Dynamic Covalent interaction	Poly(ethylene oxide)	Room temperature (RT), acidic pH	48 h	Biocompatible, cell viability, good viscoelasticity, improved mechanical stability	Acylhydrazone exchange reactions, disulfide exchange reactions	N/A	[197]
	Chitosan, Dialdehyde debranched starch (DADBS)	25 °C	<30 min	Fast crosslinking time under 30 s, tunable self-healing, excellent viscoelasticity, and mechanical properties, excellent 3D printability, obvious responsiveness to fluorescence light	Crosslinking by Schiff-base reactions between the aldehyde groups in DADBS and the amino groups in chitosan	100%	[198]
	O-carboxymethyl chitosan	RT	-	Electrostatic attraction, porous and interconnected morphology, storage modulus, excellent pH sensitive swelling properties	Schiff base reaction between the amino groups on the chitosan and aldehyde groups of crosslink agent, host-guest reaction of poly(β-cyclodextrin) with diamantine	≥97%	[45]
	Dialdehyde—modified hyaluronic acid (AHA), Cystamine dihydrochloride (Cys)	Ambient temperature	10 min	Fast crosslinking, improved mechanical properties, bioprintable, biocompatible	Schiff base reaction between the di-aldehyde groups on AHA and amino groups on Cys	~100%	[199]
	Aldehyde—functionalized surface-modified cellulose nanocrystals (a-CNCs)	RT	-	Biocompatible, injectable in situ, rapid shear thinning, cell viability, good viscoelasticity, improved mechanical stability	Schiff-base reaction between the aldehyde groups on a-CNCs and amine groups on collagen	~100%	[200]
	Lactose-modified chitosan (CTL), Boric acid, Mannitol	RT	5 min	Biocompatible, excellent viscoelasticity	Schiff base reactions between the bronic groups in boric acid and the amino groups in CTL	100%	[201]
	Triblock(ABA) copolymers with a central poly(ethylene oxide) block and terminal dithiolane blocks	25 °C	24 h,	Biocompatible, excellent Stiffness and viscoelasticity, photosensitive, mucoadhesive	The reversible ring-opening of disulfide exchange, the intracellular redox potential	N/A	[202]
	Gelatin, Dialdehyde carboxymethyl cellulose	37 °C	1 h	Excellent biocompatibility, biodegradability and non-immunogenicity, good fatigue resistance	Schiff base reaction between amino-gelatin and dialdehyde carboxymethyl cellulose	90%	[203]
	Oxidized alginate (OA), Semicarbazone (or hydrazine)	RT	10 min (or 30 min)	Biocompatibility, excellent stiffness, viscoelasticity, spreading of fibroblasts and cell adhesion, printability, non-cytotoxic	The Divalent bond between amino bonds of OA and Ca^+2^ of semi-carbazone	70% (40%)	[204]
	Acrylamide-modified chitin, Oxidized alginate	Basic pH, 25 °C	2 h	Good biocompatibility and biodegradability, excellent viability	Schiff base reactions between imine linkages amine groups of acrylamide-modified chitin and dialdehyde groups on oxidized alginate	N/A	[205]
	Chondroitin sulfate multiple aldehydes (CSMA), N-succinyl chitosan (SC)	20 °C, high moisture	2 h	Excellent viability, good biocompatibility, and biodegradability, finite inflammatory, injectable	Schiff base reactions between aldehyde groups on CSMA and amino groups on SC	N/A	[206]
Hydrogen interaction	Urethane, Urea, 2-ureido-4[1H]-pyrimidinone	RT	48 h	Excellent toughness, tensile strength, and mechanical properties	Hierarchical hydrogen bonding of urethane and supramolecular interaction	90%	[207]
	Ureido- pyrimidinone (UPy), Functionalized dextran	20 °C	10 min	Biocompatible, good mechanical properties	Ureido-pyrimidinone (UPy)- functionalized dextran	100%	[11]
	2-ureido-4[1H]-pyrimidinone (UPy), Poly(ethylene glycol) (PEG)	RT	N/A	Tunable mechanical properties, shape memory behavior. Tough	Hydrogen-bonding between UPy and PEG	N/A	[208]
	Polyurethane (PU), Tannin, Acid- modified nano tungsten disulfide	RT	12 h	Excellent mechanical strength and tensile	Noncovalent bonding connection of nano filer, interfacial hydrogen bonds between TA-WS2 and PU	100%	[209]
	Cucurbit[8]uril (CB[8]), Acrylamide, N,N′-bismethylene bisacrylamide	RT	Very fast	Good mechanical properties	Hydrogen bond and Supramolecular interaction between CB[8] and acrylamide, covalent	N/A	[185]
Ionic interaction	2-hydroxypropyltrimethyl ammonium chloride chitosan (HACC), Poly(acrylic acid) (PAAc)-Fe^3+^	70 °C	48 h	Excellent mechanical properties, tough and transparent	Both macromolecular positively charged HACC and Fe^3+^ metal ions acted as cross-linkers to form ionic bonds with negatively charged PAAc	74%	[210]
	Chitosan, Arginine (Arg), Tripolyphosphate (TPP)	RT	48 h	Tunable structural and chemical physical properties	Reaction of Polyanions of TPP and cations of amino acid arginin	N/A	[211]
	Ammonium persulfate (APS), N,N,N′,N′- tetramethylethylenedi amine (TEMED)	RT, pH ≤ 3	N/A	Anti-fatigue, good mechanical properties, time-independent healing	Positively and negatively charged groups of APS and TEMED	66–73%	[212]
Supramolecular Interaction	β-cyclodextrin modified alginate (Alg-CD), Adamantine modified graphene oxide,	RT	12 h	Injectable, good cell adhesion and differentiation, excellent mechanical properties	Guest–host interactions	100%	[213]
	Adamantane functionalized hyaluronic acid, β-Cyclodextrin	RT	12 h	Photo-cross-linkable compressible	Guest–host interactions	N/A	[157]
	β-cyclodextrin, adamantine bound by peptide tether to Hyaluronic acid	37 °C	Fast	Injectable, good cell adhesion and differentiation, excellent mechanical stability	Guest–host interactions	100%	[214]
	β-cyclodextrin-, α-bromonaphthalene functionalized acrylamide	20 °C	1 min–1 h	Injectable, excellent mechanical properties	Guest–host interactions	N/A	[215]
	β Cholic-acid, β-cyclodextrin-functionalized N,N′-dimethylacrylamide	20 °C	<1 min	Injectable, degradable	Guest–host interaction	97%	[216]
Hydrophobic interaction	Acrylamide, Octyl phenol polyethoxy ether acrylate copolymer	RT	6 days	Excellent mechanical properties, flexible	Micelles between the hydrophobic acrylates and sodium dodecyl sulfate	70%	[175]
	Cellulose nanowhiskers (CNW), Acrylamide (AM), Stearyl methacrylate, Sodium dodecylsulfat (SDS)	RT	60 min	Excellent mechanical properties, stretchable	Hydrophobic interaction of CNW and AM	100%	[217]

**Table 5:** Summary of the recent studies on self-healing hydrogels with multiple crosslinks for CTE applications.

Hydrogels (Materials)	Bonding Mechanisms	Properties	Ref.
Polyvinyl alcohol/poly(3,4-ethylenedioxythiophene)/sulfosuccinic acid	H-bonding	High water content (75 wt %)	[220]
	Crystallization	High tensile stress (~2.5 MPa)	
	Electrostatic interactions	Large elongation (>600%)	
		Conductivity (~25 mS/cm)	
Carboxymethyl cellulose/borate/gelatin	Schiff-base reaction	pH and glucose responsive	[221]
	Boronate-diol complexation		
P(urea-IL1-SPMA1)-3d IL: imidazolium-based ionic liquid SPMA: 3-sulfopropyl methacrylate potassium salt	H-bonding	Tensile strength of ~1.3 MPa	[222]
	Ionic interaction	Strain at break of ~720%	
		Toughness of ~6.7 MJ/m^3^	
Laponite^®^ nano-clay, hydroxyapatite, poly-L-arginine, sodium polyacrylate	H-bonding	-	[223]
	Electrostatic interactions		
Poly(diallyldimethylammonium chloride)/branched poly(ethylenimine)/poly(sodium 4-styrenesulfonate)/poly(acrylic acid)	H-bonding	Tensile strength: 1.26 MPa	[224]
	Electrostatic interactions	Strain at break: 2434.2%	
		Toughness: 19.53 MJ/m^3^	
Free radical polymerization of acrylic acid/acrylamide in the presence of chitosan	H-bonding	High water content (<90%)	[225]
	Electrostatic interactions	Strain at break <625%)	
		High self-healing efficiency (<88%)	
Functionalized single-wall carbon nanotube/polyvinyl alcohol/polydopamine	H-bonding	Fast self-healing ability (~2 s)	[226]
	π-π interactions	High self-healing efficiency (99%)	
		Robust adhesiveness	
Amoc (9-anthracenemethoxycarbonyl)-capped dipeptides	H-bonding	Antibacterial efficacy	[227]
	π-π interactions		
Hyaluronic acid-graft-dopamine and reduced graphene oxide/using a H_2_O_2_/HPR (horseradish peroxidase)	H-bonding	Antioxidant activity	[228]
		Photothermal effect	
	π-π interactions	Adhesive hydrogel	
		Hemostatic hydrogel	
		Conductive hydrogel	
Casein sodium salt from bovine milk/polydopamine/polyacrylamide	H-bonding	Super-stretchability	[229]
	π-π interactions	Excellent fatigue resistance	
		Rapid self-healing	
Poly (styrene-acrylic acid) core-shell nanoparticles/free radical copolymerization of acrylamide and stearyl methylacrylate	H-bonding	Excellent self-healing	[230]
	Hydrophobic interactions	Good mechanical properties	
Alginate aldehyde/poly (acrylamide)	Schiff-base reaction	Excellent self-healing and mechanical properties	[231]
	H-bonding		
Glycol chitosan/cellulose nanofiber/telechelic difunctional polyethylene glycol	Schiff-base reaction	Injectability (neural stem cells delivery)	[232]
	H-bonding		
Salicylaldehyde benzoyl hydrazone-terminal poly(ethylene glycol)/Ni^2+^	Metal–ligand coordination	Rapid self-healing Reversible pH-responsiveness	[233]
	Hydrophobic interactions		
Adamantane and β-cyclodextrin modified hyaluronic acid/methacrylated hyaluronic acid	Michael addition crosslinking (covalent reaction)	Injectability	[234]
		Rapid self-healing	
	Host-guest interactions	Cytocompatibility	
		Mechanical toughness	

**Table 6:** Comparison among fabrication methods in tissue engineering.

Method	Main Characteristic	Resulted Porosity	Cell Viability	Ref.
Freeze casting	Ceramic slurries are used in this method; then, water is evaporated. It produces pores due to formation of ice crystals.	<85%	<90%	[237]
Freeze-drying	It is an easy procedure that can be applied with natural materials such as collagen and fibers. The porosity can be improved by freezing temperature alterations and changing of the concentration of materials.	30%–80%	<90%	[238]
Solvent casting and Particle leaching	It uses casting molds to produce 3D scaffolds by polymer solution. Then, it requires leaching by using organic solvents to simplify the addition of drugs or growth factors to scaffolds.	50%–90%	75%–88%	[239]
Gas foaming	Using high-pressure carbon dioxide for expanding the polymer matrix without applying high temperature or toxic solvents. Changing pressure can also create scaled porous scaffolds.	<90%	N/A	[240]
Phase separation	Changing temperature for polymer and solvent separation results in a solid polymer due to phase separation. Finally, a desirable, homogenous, and interconnected porous scaffold is produced depending on cooling rates.	60%–98%	<98%	[241]
Electrospinning	Nanoscale or microscale fibers are produced by tuning process parameters and chemicals in this method.	80%–95%	<80%	[242,243]
Sol–gel	Colloidal metal oxides are applied traditionally to create tunable porous scaffolds in the sol–gel method with desirable chemistry. Double phasic chitosan scaffolds with a conjunction peptide have demonstrated the capability to recruit stem cells for cartilage repair.	N/A	N/A	[244]
Additive manufacturing	Extrusion methods in biomedical applications are often polymer-based and provide benefits in cost, size, and flexibility against old manufacturing methods. Both polymers and metals can be used in solid free-form sintering, while laser melting is limited to metals.	80%–90%	60%–95%	[245]

## Data Availability

The data presented in this study are available on request from the corresponding author.
